# Is ECLIA Serum Cortisol Concentration Measurement, an Accurate Indicator of Pain Severity in Dogs with Locomotor Pain?

**DOI:** 10.3390/ani10112036

**Published:** 2020-11-04

**Authors:** Adela Katalin Markovszky, Corinna Weber, Otília Biksi, Mihai Danes, Eugenia Dumitrescu, Florin Muselin, Vincenzo Tufarelli, Nikola Puvača, Romeo Teodor Cristina

**Affiliations:** 1 Faculty of Veterinary Medicine, Banat’s University of Agricultural Sciences and Veterinary Medicine “King Michael I of Romania” from Timișoara, Calea Aradului 119, 300645 Timisoara, Romania; adela.markovszky@gmail.com (A.K.M.); cris_tinab@yahoo.com (E.D.); florin.muselin@gmail.com (F.M.); 2 Laboklin GmbH & Co.KG, Steuben Str. 4, 97688 Bad Kissingen, Germany; weber@laboklin.com; 3 Hungarian Pet Physiotherapy Society, Robinson Park 41, 2337 Délegyháza, Hungary; biksioti@gmail.com; 4 Pasteur National Institute, Calea Giulesti 333 C, 60269 Bucharest, Romania; mihai.danes@pasteur.ro; 5 Department of DETO, Section of Veterinary Science and Animal Production, University of Bari “Aldo Moro”, 70010 Bari, Valenzano, Italy; vincenzo.tufarelli@uniba.it; 6 Department of Engineering Management in Biotechnology, Faculty of Economics and Engineering Management in Novi Sad, University Business Academy in Novi Sad, Cvećarska 2, 21000 Novi Sad, Serbia; nikola.puvaca@fimek.edu.rs

**Keywords:** dog, pain level, cortisol value, significance, ECLIA

## Abstract

**Simple Summary:**

Serum cortisol level reflects the activity of stress axis, ethological alterations, acute and chronic pain, life quality, or psychogenic stress. Although it is stated that stress can produce a measurable influence on the cortisol level, a certified value of this pain biomarker in dogs was not generally accepted yet. This interdisciplinary research emerged from the need for information in this field since not many studies were focused mainly on comparative analysis. We consider this field as a hot topic with various possible applications. Our survey is a methodological study within the fields of behavior and veterinary sciences, being relevant for the dog’s pain assessment. Results are having the guarantee of the high standard analysis of serum specimens, and using the updated Cortisol assay, with serum cortisol determined by Electrochemiluminescence immunoassay. We consider that our work could refresh the information in this field. Yet, an area of interest, specific pathology and pain particularities in a dog, being studied more and more in the last decade. What we can say is that serum cortisol limits cannot be adopted as a single and accurate pain marker in dog species, our study confirming these values, as non-conclusive for the assessment of the real pain levels in the dogs.

**Abstract:**

The purpose of determining serum cortisol level is to reflect the activity of stress axis, ethological alterations, acute and chronic pain, life quality, or psychogenic stress. Although it is stated that stress can produce a measurable influence on the cortisol level, a certified value of this pain biomarker in dogs was not generally accepted yet. This study aimed to investigate if serum cortisol measured follows allopathic treatments only, or it is associated with physiotherapy, point out pain level in dogs with orthopedic disease, which could reveal the healing progress. The diagnostic identified: hip dysplasia, cranial cruciate ligament rupture, or intervertebral disc disease. Ortolani and Barden tests, together with clinical examination, drawer sign, and tibia compression test, were done in dogs exhibiting postures, and motion alteration, and X-Ray confirmed. A total of 30 dogs were grouped in healthy (*n* = 10) and pain groups (*n* = 20), the blood sampling is done at the beginning of the investigation, and after ten days of the study. Dogs were handled in two ways: G1—treated with Nonsteroidal Anti-Inflammatory Drugs (NSAIDs) only and respectively, G2—by therapy and physiotherapy. The analysis was performed on a Roche Cobas Analyzer (Roche, USA), serum cortisol being determined by Electrochemiluminescence immunoassay (ECLIA), and statistics using ANOVA, following Tukey’s Multiple Comparison Test. The results revealed that, out of ten specimens in the Control group, nine were within the normal limits: 5–65 ng × mL^−1^ (24.76 ± 19.48678), and one sample under the set limit. In G1, it was observed that the plasmatic P1 values were below the levels of P2, in six situations. In G2, although the status of all subjects improved radically with the removal or evident reduction of pain, confirmed clinically and imagistically, the P2 values in five dogs were higher than the initial P1 values, and in contradiction with the observed clinical reality. Comparing results, the mean difference in G1 was 0.41, and in G2 = 2.54, with an SD for G1 = 13.38, and G2 = 16.66, registering moderate development. Standard deviation illustrated that the values of treated groups were highly spread throughout the interval, and the serum cortisol assay did not generate significant statistical differences between groups in our case. This inferred the doubt whether the used detection method or values registered correctly indicates the pain levels in dog species.

## 1. Introduction

Locomotor pain is one of the most common diagnoses clinicians make in daily practice. Lately, it has become more and more important for pet-owners to ensure that pets have a good life-quality. For reasons of animal welfare and also to ensure a rapid recovery of dogs suffering from locomotor pain, it is essential to adequately treat acute or chronic pain. Furthermore, pain levels should be taken into consideration when choosing the proper analgesia and physiotherapy [1]. The pain alleviation techniques in dog species have been rapidly increasing in the last decade. From the clinical point of view, the efficiency of animal recovery and the rapid improvement of the condition, life quality, and patient mobility, generated the implementation of new methods and allopathic pain treatment protocols [2,3,4,5,6]. 

The pain assessment methods can be subjective or objective. Examples for subjective methods are pain scales, such as the Helsinki Chronic Pain Index (HCPI), Glasgow Composite Measures Pain Scale (CMPS), visual analogue scales (VAS) or simple descriptive scores (SDS) [1]. Gait analysis would be a more objective method, but also has its limitations [7,8]. Cortisol measurement remains an important method to assess stress associated to pain in dogs, amongst other biomarkers like catecholamine or physiological markers like heart rate, blood pressure or respiratory rate. The main functions of cortisol are the involvement in gluconeogenesis and the anti-inflammatory reaction, and most importantly, in the stress mechanism [4]. The stress reaction is coordinated by the paraventricular nucleus of the hypothalamus, stimulating the secretion of other hormones such as vasopressin, prolactin, and catecholamine [8]. Every initiation of the sympathetic nervous system can cause cortisol elevation. Though it is acknowledged that acute stress has a measurable impact on cortisol levels in the body, the effects of chronic pain have failed to be confirmed [8,9,10].

The purpose of determining serum cortisol levels is to reflect the activity of the stress axis, ethological modifications, acute and chronic pain, life quality, or psychogenic stress. Cortisol can be assessed from serum, hair or saliva. Researches have shown that the collection of saliva samples and the detection of salivary cortisol provides results comparable to the serum cortisol level measurements [4,8,11,12]. Moreover, it was reported that cortisol accurately indicated the chronic stress in hair or fur when non-invasively collected, due to the cortisol involvement in the sebaceous and sweat excretion during hair growth [8,12,13,14,15].

Researchers have tried to establish cut-off values for cortisol levels that correlate with pain in dogs. According to the study conducted by Feldsien et al. [16], a value of serum cortisol smaller than 1.6 µg × dL^−1^ is improbably associated with articular pain in dogs. Values greater or equal to 1.6 µg × dL^−1^ indicate dogs suffering from pain but also include some false-positive results [16]. In the case of pain due to canine gastritis, average serum cortisol of the dogs without any pain was 0.25 ± 0.05 µg × dL^−1^, and of those with gastric pain was 5.59 ± 1.90 µg × dL^−1^, with this significant difference suggesting that serum cortisol can potentially be used as a pain marker [17]. On the other hand, as reported by Reese et al. [2] and Egger et al. [3], the level of serum cortisol correlates weakly with pain diagnosed using subjective methods of postoperative pain evaluation after repair of cranial cruciate ligament rupture in dogs [2,3].

Serum cortisol is considered a useful biomarker, especially in the detection of acute, and less, of chronic pain, mainly in combination with additional subjective methods such as behavioral analysis [1,18,19,20,21]. In chronic pain, a decrease of serum cortisol level is possible because of the negative feedback generated by the prolonged secretion of the glucocorticoid [22,23]. Additionally, the animals tend to get adapted to the blood sampling protocols, and it is possible that at the second sampling, the psychogenic stress no longer affects the serum cortisol level. Hence, cortisol blood levels could be lower [1,20,21].

Despite multiple possibilities, a generally validated pain biomarker in dogs has not yet been accepted. Although questionable, the cortisol value remains a sole pain indicator used; however, other biomarkers are studied [1,20]. Furthermore, there are many new rehabilitation techniques combined with drug treatments, where the serum cortisol value measured before and after treatments, is recommended to be employed as a pain indicator [21,24].

The purpose of the study was to establish if the serum cortisol values measured following allopathic treatments alone or following allopathic treatments associated with physiotherapeutic techniques effectively mirror the pain severity in dogs with orthopedic disease, to reflect the real clinical progress and indicate the best combinations of therapy.

## 2. Materials and Methods

In the interval between the year 2018 and 2019, a total of 30 dogs, *n* = 10 (healthy), and *n* = 20 (with confirmed pain of the locomotor system (limbs, vertebral column, or combined) were observed, respectively. Dogs were regular patients of two veterinary clinics, Gesundheitszentrum für Kleintiere, Passau, Germany, and Praxis Dr. Pauli, Waldkirchen, Germany. To perform this study, no special approval was needed because therapy was in accordance with the legislation; all the owners had been previously informed, and all consented to the therapeutic and recovery protocols used for their dogs. All therapeutic protocols were routine ones and would have been applied to these dogs also outside this study. All dogs were already dewormed, and the vaccine status was verified for each patient. Dogs included in the study are presented in Table 1.

The treatment of dogs with pain symptoms (Group 1), was performed using the following oral or injectable Nonsteroidal Anti-Inflammatory Drugs (NSAID) active substances:

- Robenacoxib (Onsior, Elanco Tiergesundheit AG, Germany): 1.0–2.0 mg × kg bw^−1^
- Firocoxib (Previcox, Merial, France): 5.0 mg × kg bw^−1^
- Meloxicam (Melosus, CP-Pharma, Germany): 0.2 mg × kg bw^−1^
- Carprofen (Carprotab, CP-Pharma, Germany): 4.0 mg × kg bw^−1^

The NSAID therapeutic protocol was individually adapted to each dog, following the animal’s body weight, possible adverse reactions, the owner’s preferred medication route (oral or injectable). The most suitable anti-inflammatory drugs were administered once per day for ten days, and the blood samples were collected at the start, and once the treatments were finalized (Table 2), always in the timeframe between 9:00 and 13:00.

Dogs exhibiting clinical pain, whose owners agreed with physiotherapy and treatment (Group G2), were clinically analyzed and treated accordingly. The diagnosis was determined, and corresponding drug therapy was prescribed depending on the individual physiotherapy protocol (Table 3).

To limit the stress onset, an owner or an assistant in the owner’s presence executed the dog restraint. The hair trimmer use was avoided. As a result of the established diagnostics and therapeutic protocols, three separate dog categories were identified for blood sample collection:

### 2.1. Dogs with Pain—Requiring Anesthesia

After the clinical examination, an intravenous indwelling cannula was placed in the cephalic vein of the dog. Before the administration of anesthetics or any other active substances, the initial blood samples were collected in red sterile vacutainer tubes. 

Then anesthesia was administrated with following protocol: Injection anesthesia Medetomidine 1 mg × mL^−1^ (20–80 µg × kg bw^−1^) + Propofol 1% (induction: 0.4 mL × kg bw^−1^; maintenance 0.2 mg × kg bw^−1^); afterwards Atipamezol 5 mg × mL (200 µg × kg bw^−1^). A total of 5 dogs underwent anesthesia. 

After ten days of NSAID’s medication, alone or associated with physiotherapy, post-treatment blood samples were collected. To minimize the venipuncture stress, sampling was performed in a separate quiet room where no medical examinations or interventions were carried out at the time.

### 2.2. Dogs with Pain—Not Requiring Anesthesia

In this group, dogs did not have the intravenous catheters placed in the cephalic vein, and the initial blood samples were obtained in sterile red vacutainer tubes, before the administration of anesthetics or any other drugs, by direct venipuncture with G20 yellow needles. As for the first group, after ten days of NSAID administration, alone or associated with physiotherapy, blood samples were collected.

### 2.3. Healthy Dogs—Without Pain

Blood was collected after a single direct venipuncture. These dogs were previously (a day before blood sampling) clinically examined to minimize the psychogenic stress generated by the examination.

### 2.4. Determination of Serum Cortisol

Blood samples were left to rest for 15 min protected from the light, and later they were centrifuged at 3000× *g* at room temperature for 5 min in an EBA 20 centrifuge (Hettich, Darmstadt, Germany). The serum samples, at least 250 µL, aspired with an automatic pipette were stored in sterile tubes positioned vertically at −20 °C, kept maximum for three months to preserve the stability of cortisol. The frequency of sample dispatch was dependent on the sampling date, but the maximum storage period was never reached.

The analysis of serum specimens was performed at certified Laboklin Labor für Klinische Diagnostik GmbH & Co. KG., in Bad Kissingen, Germany, using the Cortisol assay (Roche Diagnostics, Indianapolis, IN, USA). Sample analysis was performed by Roche Cobas 702 Analyzer (Roche Diagnostics, Indianapolis, IN, USA), and serum cortisol was determined using Electrochemiluminescent Immunoassay (ECLIA), with testing kits provided by Roche (Roche Diagnostics, Indianapolis, IN, USA). Samples submitted for testing were incubated with a biotinylated antibody and with a cortisol derivate marked with ruthenium, with the binding sites of the marked antibody occupied by the cortisol from the samples according to the concentration, and ruthenilated haptens. Micro particles coated with streptavidin were added, and by the magnetic effect, formed complex was attached to the solid phase. Next, the unfixed forms were separated, and the electric voltage was applied to induce chemiluminescence.

### 2.5. Statistical Analysis

Values were expressed as mean ± Standard Error Mean (SEM). The evaluation of the variance between groups was ascertained using the two-way ANOVA, for samples, with Tukey’s multiple comparison test, considering that, the differences are statistically provided when *p* < 0.05, or less. The applied software was Graph Pad Prism 6.0 for Windows (Graph Pad Software, San Diego, CA, USA).

## 3. Results

The classification of pain level was based on the pain intensity, and it was performed after the complete clinical and imagistic examination (but without taking into consideration the level of pain suggested by the owners). Accurate diagnostic techniques were utilized for the identification of hip dysplasia, cranial cruciate ligament rupture or intervertebral disc disease: Ortolani and Barden test together with the clinical examination, drawer sign, and a tibia compression test were done in dogs exhibiting pain, different postures and motion alteration also confirmed by the X-ray. Clinical observation of healthy dogs did not reveal significant modifications.

**Figure 1:**
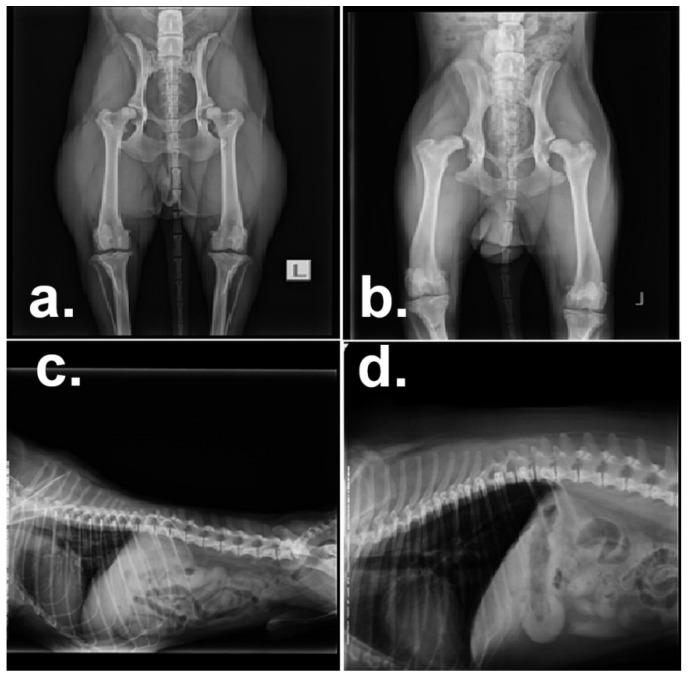
Pathological entities found in dogs exhibiting pain. (**a**). Hip dysplasia Mixed breed Labrador Saint Bernard, grade 2–3 (right); grade 1 (left). (**b**). Hip dysplasia Labrador retriever; femoral head: luxation (right); sub-luxation (left). (**c**). Suspicion of intervertebral disc disease narrowing Rusky toy (space L4–L5). (**d**). Intervertebral disc disease narrowing Mops (spaces T6–T7 and T10–T11).

In Figure 1, four suggestive pathological entities found in dogs exhibiting pain are presented.

Table 4, and the supplementary Figure S1 are showing the measured serum cortisol levels, and the evolutions for all dogs studied.

In the group of healthy dogs, nine out of ten examined samples were within the established limit, with values registered between 5 and 65 ng × mL^−1^ (SD = 24.76 ± 19.48678), and one registered sample was below the set limit. 

In the group of dogs which received only drug treatment (G1) in the case of six dogs, no. 4, 5, 7, 8, 9, and 10, it was observed that the registered pain line values P1 were even below the levels of P2. As the literature shows, serum cortisol is influenced by stress associated with blood sampling, but also by the time of the unpleasant, painful stimulus action on the animal [21]. This possibly could explain the aforementioned inconsequent values found in dogs, though appropriate precautions to eliminate all added stress at the sampling moment were considered. 

Furthermore, it was determined that in persistent pain cases, cortisol could provide negative feedback to the hypothalamic-pituitary axis. Therefore, the P1 discerned values could be falsely lower compared to the real pain level. On the other hand, authors demonstrated that individual variation of cortisol and other hormones in plasma of dogs with chronic pain are usually very high and unpredictable, so that these biomarkers are not as useful as validated pain scales for measurement of the chronic pain [11,20,25].

In the drug and physiotherapy group (G2), even though the status of all patients improved significantly, with the total disappearance or apparent reduction of pain signs, confirmed clinically and imagistically, the registered P2 values in dogs no. 2, 5, 6, 7, and 9, were higher than the initial P1 values, and this was in contrast with the clinical reality. In this group, the NSAID treatment for ten days, associated with physiotherapy, the patient stress at the sampling and negative feedback, all influenced the serum cortisol values. It should be mentioned that dogs treated with physiotherapy developed a high amount of trust towards the physiotherapist, and they had a much more relaxed attitude, as also revealed in the field literature. Still, at the time of blood sampling, their state was automatically altered [19,24].

In order to compare the two groups the difference between P1 (initial value of serum cortisol) and P2 (final value of serum cortisol) was calculated. Comparing the results of the two treatment groups, the mean difference in the drug therapy group (G1) was 0.41 and in the drug therapy and physiotherapy group (G2) was 2.54. The SD for G1 = 13.38, and G2 = 16.66, are indicating a milder evolution in G2. 

Consequently, the serum cortisol levels in the drug therapy group, before and after treatment, placed closer to one another, in comparison with those of the drug therapy and physiotherapy group. Standard deviation illustrated that the values of both groups were greatly spread throughout the interval, but they are somewhat similar. Serum cortisol analysis did not generate significant statistical differences between the pain groups analyzed in our study, and this has raised the uncertainty of whether the detection method used, or the registered values, genuinely reflect the degree of pain. 

**Figure 2:**
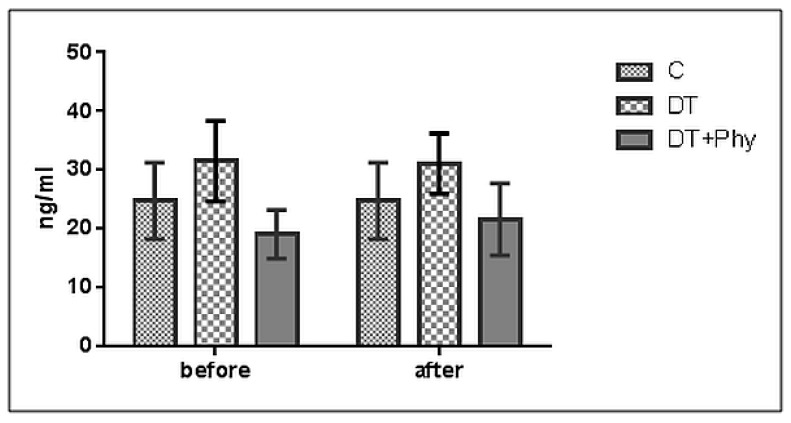
Mean values before and after treatments/dog groups (C = Control, DT = Drug therapy, DT + Phy = Drug therapy + Physiotherapy) revealing no significant statistical correlation between pain/dog groups.

In Figure 2, the ascertained serum cortisol levels revealed no significant statistical correlation between pain/healthy dog groups.

## 4. Discussion

This study attempted to objectively and comparatively evaluate the efficiency of the antalgic therapy alone and associated with physiotherapy in dogs by measuring the cortisol serum levels in apparent pain, expecting that the obtained results would indicate a positive correlation between the serum cortisol levels and the severity of pain. Unluckily, a statistically significant result was not achieved, most probably due to the blood sampling and other procedures performed on the patients, generating the idea that the development of new alternative methods for blood sample collection would be beneficial in the assessment of dog pain severity and its stages “fine-tuning”.

In this study, all dogs suffered from the same pain type, respectively, the locomotor system (the limbs, vertebral column, or combined), clinically and para-clinically confirmed, so is circumscribed to this source of pain. Furthermore, the study was completed in a normal veterinary clinic environment, and not in a full experimental facility. This meant that patients and their records were obtained as usual in a clinic (considering that these values are really valuable for practitioners); and this could have influenced the results, knowing that cortisol value varies throughout the day [4]. 

However, even though authors recognized the pulsatile cortisol secretion, recently, it was also observed that, in canine species, this process occurs while not respecting the circadian rhythm, in contrary to other species analyzed [21,24].

Intact bitches have higher cortisol concentrations. They tend to react more intensively to stress factors, which is called HPA-hyper reactivity. In our study we could not detect this phenomenon, but this could be due to the small number of females included in the study [25]. 

Nevertheless, as other more and more authors observed, it is essential to mention that the results of immunoassays can be affected by the cross-reactivity of additional steroids and the treatment with medication containing glucocorticosteroids, and depending on the level of cross-reactivity with the assay used, the obtained results may be altered [26,27]. Distinctly, prednisone and his specific metabolites are chemically analogous to serum cortisol and may heavily conflict with cortisol values determined by immunoassay methods [28,29]. 

Cortisol levels may be influenced by anesthetics used for sedation of the dogs. However, cortisol levels after propofol administration seem to restore to premedication levels after 24 h, whereas medetomidine seems to have no influence on them. Atipamezole triggers a faster recovery to premedication levels of cortisol [30,31,32].

Nowadays, various methods of measuring cortisol, used as a biomarker of stress and pain, are known, and all of them carry imperfections [33]. In the majority of earlier investigations, cortisol values were measured in biological specimens by immunoassay techniques. Firstly, radio-immunoassay (RIA) was used [26]. It was replaced by enzyme-linked immunosorbent assay (ELISA) [29], automated electrochemiluminescent assays (ECLIA) [28], and the most modern liquid chromatographic methods coupled with mass spectrometry detection (LC-MS/MS) [27,34].

Some methods are assessing the short-term variations, while others are focusing on the long-term changes. For the chronic pain assessment, it would be clinically more useful to be able to determine the long-term variations of cortisol, but measuring this parameter could be misleading since multiple cut-off values were shown in the literature, depending on the method used, and many clashing opinions on this subject emerged in the last decade [4,7,21,24].

Other modalities to measure chronic and acute pain include measurement of prolactin, serotonin, catecholamines or evaluation of cardiac frequency, arterial pressure. Subjective methods of pain measurement are questionnaires, such as Helsinki Chronic Pain Index (HCPI) or Glasgow Composite Measures Pain Scale Short Form. These two types of questionnaires were used in our study to simultaneously evaluate pain levels. We therefore grouped the dogs differently according to acute and chronic pain shown and included/excluded some patients. Pain scale results correlated better with our clinical findings. This information will be published in another article accepted. An Objective pain measurement method would be gait analysis. The combination of various methods should be taken into consideration [7,8,24,25,34,35].

However, a new methodology in this domain is developing, and the non-invasive methods of the cortisol evaluation represent a reliable alternative. For example, as used in human studies, employing hair cortisol as an indicator of chronic stress could constitute a useful tool in the veterinary field [36]. Additionally, Ouschan et al. [37] identified a median cortisol concentration (1.5 ng × g^−1^) in a healthy dog’s hair and elevated cortisol concentrations in the hair of dogs with hyperadrenocorticism [37].

Moreover, there is a method presented by Mack and Fokidis [12], where cortisol can be determined using a dog’s claw, and it also correlates with hair cortisol levels. This is a method that could prove an association with chronic pain [12]. Furthermore, it has been stated that measuring saliva cortisol levels can deliver variations in results. Thus it is still a tolerated cortisol measurement method in dogs, reducing psychogenic stress [11,13,15]. 

From the last decade’s literature, we recognize that hair cortisol levels correlate positively with saliva cortisol levels, while hair cortisol determinations show variation linked to coat color [14,36,37,38].

According to Lane et al. [29], the determination of serum cortisol in a reference laboratory through validated CLA is a suitable method for the evaluation of serum cortisol concentration in dogs in cases where the ELISA test gives equivocal results [29]. Still, Radisavljević et al. [24] obtained no statistically significant difference in serum cortisol concentration of dogs tested with ELISA after transportation. Yet, the serum cortisol concentration 24 h after transportation tended to be lower than immediately after transportation [24].

Although serum cortisol concentration has currently proven to indicate pain in dogs at a cut-off level of 6.6 µg × dL^−1^, but following our evaluation and methods used, this value has shown to be unreliable and in some cases overestimated, with the clinical reality often contradicting this value [21]. 

Errors can be reported even with ECLIA, our method used, considered suitable for this study, and other newer techniques of serum cortisol detection (most probably due to possible interferences) [28], and because of the several non-invasive alternative matrices analyzed, like saliva [13,15,33], hair [36,37,38], nails [12], which may not fall within this limit. In this respect, estimating the pain levels creates the need for a unanimously accepted cut-off value. This value being in correlation with the analytical method and matrix used, together with the clinical evidence, are all setbacks to be solved. 

Our results showed that individual variation of cortisol in the serum of dogs from different breeds was too significant to express this parameter as a uniform and useful biomarker of pain in dogs. In the study of Davila et al. [5], statistical differences between serum cortisol values in three groups of dogs with acute pain after osteotomy were not found to be significant either [5]. Another study could not show significant statistical differences between serum cortisol levels in groups suffering from pain following ovariohysterectomy [6]. 

To establish the best therapeutic combinations between drugs alone or when possible to associate them with physiotherapy (until a unanimously accepted cortisol detection method with clear limits will be ascertained for dogs), the clinical and investigational means, associated with the clinician’s experience, will remain to be the most indicative. In support of that, we observed that drug therapy combined with physiotherapy favorably influenced pain healing in all cases. However, the serum cortisol values did not reflect this objective reality.

## 5. Conclusions

In daily veterinary practice, serum cortisol limits cannot be adopted as an exclusive and accurate pain marker in dog species; our results consider these values as non-conclusive for the assessment of real pain levels in dogs. Therefore, the serum cortisol margin value cannot be used as a “crucial”, but only as an “indicative”, pain biomarker in dog clinical evaluation.

## Figures and Tables

**Table 1:** Dogs of different categories included in the study.

No.	Breed	Age	Weight	Sex
14	Border Collie (P)	1.5 years	25 kg	m
6	Golden Retriever (P)	8 years	36 kg	nm
10	Labrador Retriever (P)	11 years	48 kg	nm
13	Labrador Retriever (P)	4 years	30 kg	nf
19	Malinois (P)	7 months	12 kg	
11	Mixed breed German Shepherd (P)	12 years	35 kg	nf
12	Mixed breed German Shepherd (P)	10 years	35 kg	nf
17	Mixed breed Husky (P)	4 years	20 kg	f
5	Mixed breed Labrador (P)	10 years	26.7 kg	nm
8	Mixed breed Labrador (P)	10 years	15 kg	nm
15	Mixed breed Labrador (P)	3 years	23 kg	m
1	Mixed breed Labrador Saint Bernard (P)	2 years	19 kg	nm
16	Mixed breed Pitbull (P)	5 years	30 kg	nm
18	Mixed breed Pitbull (P)	1 year	13 kg	m
3	Mixed breed Romanian mioritic Shepherd (P)	7 years	32.7 kg	nf
2	Mops (P)	8 years	11.6 kg	nm
1	Pudel (P)	9 years	8.1 kg	m
4	Rusky toy (P)	2 years	3 kg	m
9	Saint Bernard (P)	2 years	50 kg	f
7	Terrier Mix (P)	11 years	10 kg	nm
20	Westie (P)	14 years	10 kg	f
23	Australian Shepherd (H)	4 years	25 kg	nf
27	Border Collie (H)	11 years	25 kg	nm
21	Jack Russel Terrier (H)	14 years	16 kg	nf
22	Jack Russel Terrier (H)	11 years	17 kg	nm
28	Labrador Retriever (H)	1 year	29.8 kg	nm
24	Mixed breed (H)	2 years	18.9 kg	nf
29	Mixed breed (H)	7 months	6.2 kg	nf
30	Mixed breed (H)	2 years	9.2 kg	nm
26	Parson Jack Russel (H)	15 years	7 kg	nf
25	Spitz (H)	7 months	6 kg	f
Average age ±SD (standard deviation) and average body weight ±SD				
Category	Healthy dogs (*n* = 10)	Dogs with pain (*n* = 20)		
Age (months) ± SD	72.125 ± 59.41245	74.11111 ± 56.29428		
Body weight ± SD	19.625 ± 7.595817	24.24444 ± 14.13632		

nm—neutered male; nf—neutered female; f—female; m—male; H—healthy; P – pain.

**Table 2:** Nonsteroidal Anti-Inflammatory Drugs (NSAID) administration protocol.

No.	Breed/Sex	Clinical Examination/Diagnose	Administered Drug
1	Mixed breed, female	Lameness, severe acute pain in the knee joint, right leg, ligament injury	Onsior (robenacoxib) 20 mg × mL^−1^ injectable 0.65 mL, afterwards Onsior Tabl. 10 mg 1 × 1/day
2	Mixed breed Mioritic shepherd, female	Lameness, moderate acute pain in the knee joint of the left leg, articular effusion, reduced patellar crepitation with a slight possibility of lateral luxation	Onsior 20 mg × mL^−1^; inj. 3.30 mL, afterwards Previcox (pirocoxib) Tablets 227 mg 1 × 1/day
3	Golden Retriever, male	Positive drawer sign, moderate pain, cranial cruciate ligament rupture of the left leg, (surgical intervention)	Onsior 20 mg/mL inj. 3.80 mL 2 days, afterward Previcox (firocoxib) Tabl. 227 mg 1 × 1/day
4	Pudel, male	Lameness of the right hind leg, reduced to moderate pain, positive drawer sign, cranial cruciate ligament rupture, medial patellar luxation (surgical treatment)	Onsior 20 mg × mL^−1^ inj. 0.90 mL 2 days, Onsior 20 mg Tabl. 1 × 1/day
5	Terrier Mix, male	Lameness, moderate pain, cranial cruciate ligament rupture in the right leg, (surgical treatment)	Melosus (meloxicam) 5 mg × mL^−1^ 0.4 mL injectable 2 days, afterwards Carprotab, (carprofen) 50 mg 1 × 1/day
6	Border Collie, male	Chronic pain at the lumbar vertebral column, stiff gait after acutisation	Previcox Tabl 227 mg 1 × 1/day
7	Mixed breed German shepherd, female	Positive drawer sign, lameness in the posterior right leg, moderate pain, cranial cruciate ligament rupture	Melosus 5 mg × mL^−1^ 1.4 mL injectable 3×, afterward Previcox Tabl. 227 mg 1 × 1/day
8	Labrador, male	Moderate chronic pain in the lumbar vertebral column	Melosus 5 mg × mL^−1^, 1.9 mL injectable 1×, afterwards Previcox, Tabl/227 mg 1½ × 1/day
9	German shepherd, female	Lameness of the right anterior leg, articular effusion in the elbow joint, severe pain, polyarthritis	Melosus, 5 mg × mL^−1^ 1.4 mL, injectable 1×, afterward Previcox, Tabl. 227 mg 1 × 1/day
10	Malinois, female	Lameness of the right posterior leg, acute pain, ligament injury	Melosus, 5 mg × mL^−1^ 0.5 mL injectable 1×, afterward Previcox, Tabl. 227 mg 0.5 × 1/day

**Table 3:** Physiotherapy protocol used.

No.	Breed/Sex	Clinical Examination/Diagnose	Administered Drug
1	Rusky toy, male	Temporary paraplegia of 15 min, afterward recovery to a healthy state. The painful vertebral column, radiology examination: narrowing of the intervertebral space L4–L5. Diagnosis: Disc injury. Patellar Luxation (already surgically treated) in the left leg. Physiotherapeutic evaluation: low back at the lumbar vertebral column level, negative Kibbler, muscular atrophy in the hind legs, can tremble completely.	Electrotherapy (ET)—amplitude modulated current 4000 Hz, massage, reduction of muscular atrophy by exercise. ET modulated in the frequency interval of 100–250 Hz, 11 mA; Exercises: sit-lay down-come 3–4×/day × 4–5 times. After two sessions: Kibbler positive. Physiotherapeutic treatment for ten days. Drug treatment: 7 days with Onsior, 20 mg × mL^−1^ injectable 0.30 mL, afterward Onsior, Tablets 6 mg 1 × 1/day
2	Mixed breed Labrador, male	Severe acute pain at the cervical spine level during rotation to the left and, in extension, exaggerated proprioception at the lower limbs—moderate chronic pain at the lumbar spine level with progressive worsening. Physiotherapeutic evaluation: Kibbler negative, cannot tremble, attentive, and cautious walk.	Electrotherapy (ET)—amplitude modulated current 4000 Hz, massage ET 100–250 Hz, 12 mA at C7-T1 and T13-L1. After two sessions: Kibbler positive can tremble. Physiotherapeutic treatment for ten days with a recommendation to a life-long continuation at a rate of 1 treatment every 7–14 days, depending on the evolution. Drug treatment: 7 days with Onsior, 20 mg × mL^−1^ inj. 2.70 mL 1 day, afterward Previcox, Tabl. 227 mg 1 × 1/day.
3	Mixed breed, female	Intermittent lameness of lower limbs, extension, and rotation of both hind limbs reduced. Physiotherapeutic evaluation: Kibbler positive, trembles wholly but rarely, back in persistent contraction.	Electrotherapy (ET)—amplitude modulated current 4000 Hz, Hydrotherapy. ET 100–250 Hz, 20 mA at the hips. Hydrotherapy with water reaching the hips: Session *S1*: 105 m walk, 1× pause with water reaching knees. *S2*: 0.6 km/h slow walk, 163 m. *S3*: identical *S4*: 0.8 km × h^−1^, 345 m. *S5*: identical. *S6*: 0.7 km × h^−1^, 467 m. *S7*: identical. *S8*: 0.8 km × h^−1^, 530 m. *S9*: 0.8 km × h^−1^, 660 m. *S10*: identical, but after 8 min with a cuff.
4	Mixed breed Labrador-Sain-Bernard, male	Hip dysplasia grade 2–3 on the right, grade 1 on the left, moderate pain in the spine. Physiotherapeutic evaluation: mild chronic pain with activation at T13-L1 level.	Electrotherapy (ET)—amplitude modulated current 4000 Hz, hydrotherapy, laser. Laser F-L: NogC, 2J 10 sessions. ET 100–250 Hz, 16 mA at the spine T13-L1. ET 1-250, 4.4 KHz, 14.5 mA the whole spine. Hydrotherapy with hip-high water level: Session *S1*: 100 m walk, 2× break with knee-high water level. *S2*: 0.6 km/h slow walk, 170 m. *S3*: identical *S4*: 0.8 km × h^−1^, 425 m. *S5*: identical. *S6*: 0.7 km × h^−1^, 470 m. *S7*: identical. *S8*: 0.8 km × h^−1^, 540 m. *S9*: 0.8 km × h^−1^, 700 m. *S10*: identical.
5	Mops, male	Intermittent ataxia, intermittent lameness, intermittent paraplegia. Spontaneous recovery after a few seconds. Moderate to severe pain at the thoracolumbar transition site. Delayed proprioception in the right hind leg. Radiological diagnosis: narrow intervertebral spaces T6-7, T10-11. Diagnosis: discopathy	Electrotherapy (ET)—amplitude modulated current 4000 Hz, massage, hydrotherapy, laser. ET 100–250 Hz, 16 mA, 10 sessions. Laser F-L: NogE, 3J. 1 session. Hydrotherapy with hip-high water level: Session *S1*: habituation to walking (reticent patient), *S2*: 0.6 km × h^−1^ slow walk, 94 m, *S3*: 100 m, hypermetrics of posterior limb, *S4*: 170 m *S5*: identical, *S6*: 0.6 km × h^−1^, 225 m, *S7*: 188 m walk, *S8*: 0.6 km × h^−1^, 200 m, *S9*: 0.6 km × h^−1^, 200 m, *S10*: 220 m 26 min.
6	Labrador Retriever, male	Femoral head luxation right leg and subluxation left leg, hip dysplasia (femoral head resection on the right side). Moderate to severe pain. Physiotherapeutic evaluation: 10 days after surgical treatment. Stands on all four legs at walk and trot, stabilizes his position at the sacrum.	Electrotherapy (ET)—amplitude modulated current 4000 Hz, Massage, Hydrotherapy ET 100–250 Hz, 30 mA. ET 1–250 Hz, 22 mA. pROM exercises. Hydrotherapy with hip-high water level: Session *S1:* 11 m walk, 3× break with water reaching knees. *S2*: 0.6 km × h^−1^ slow walks, 61 m, 10 min, 3× break. *S3*: identical. *S4*: 0.6 km × h^−1^, 137 m, 20 min. 3× break. *S5*: identical, *S6*: 0.6 km × h^−1^, 140 m, 20 min. *S7*: 188 m without a break. *S8*: 0.8 km × h^−1^, 403 m, 32 min. *S9*: 0.8 km × h^−1^, 500 m, 40 min. *S10*: 550 m, 40 min.
7	Mixed breed Pitbull, neutered male	Moderate pain at the lumbar spine level. Physiotherapeutic diagnosis: Kibbler negative, partial trembling.	Laser NogE, Frequency 2.400 Hz, amplitude 80%, 240 s. Drug treatment ten days with Melosus, 5 mg × mL^−1^ 1.2 mL injectable 1× then Previcox, 1 × 1/day 9 days
8	Mixed breed Husky, female	Moderate pain at the cervical spine level. Neck rotation to the right is not entirely possible because of the pain.	Massage Drug treatment ten days with Metacam 5 mg × mL^−1^ 0.8 mL injectable 1× then Previcox, 0.5 × 1/day nine days
9	Saint Bernard, female	Moderate pain in the right hip, intermittent lameness during the increased effort, extension of the right hind leg not utterly possible in comparison with the left leg. Presumptive diagnosis: Hip dysplasia. Recommendation: Radiographic examination under anesthesia	pROM exercises, Drug treatment Previcox, 227 mg 1½ × 1/day, ten days
10	Mixed breed Pitbull, male	Moderate pain at the lumbar spine level, careful gait, slower gait, Kibbler impossible, trembles incompletely.	pROM exercises, massage, laser. Laser NogE, Frequency 2.400 Hz, amplitude 80% and 240 s. Drug treatment ten days Previcox, 227 mg 0.5 × 1/day

**Table 4:** Serum cortisol levels before and after treatments/dog groups given as ng × mL^−1^.

No. Crt.	Healthy Patients	Patients Treated with Drug Therapy Only		Patients Treated with Drug Therapy and Physiotherapy Methods	
		Before Treatment	After Treatment	Before Treatment	After Treatment
1.	7.5	54.8	27.5	18.3	10.1
2.	11.3	75.4	62.7	5.9	9.6
3.	7.5	43.1	28.0	41.6	15.6
4.	32.2	14.8	31.8	25.8	14.2
5.	22.2	37.3	48.6	41.6	64.8
6.	55.5	8.5	6.8	13.6	48.4
7.	57.7	12.1	15.0	9.2	14.4
8.	0.9	13.6	24.8	11.6	7.9
9.	39.7	31.3	41.5	10.1	22.1
10.	13.1	24.2	24.3	13.2	9.2

## Supplementary Materials

The following are available online at https://www.mdpi.com/2076-2615/10/11/2036/s1, Figure S1: Evolution of serum cortisol in dogs used in the study.
