# Supplementary figures and images for: Is ECLIA Serum Cortisol Concentration Measurement, an Accurate Indicator of Pain Severity in Dogs with Locomotor Pain?
Source: Animals (Basel). 2020 Nov 4;10(11):2036. doi: 10.3390/ani10112036 (PMC7694258; doi:10.3390/ani10112036)

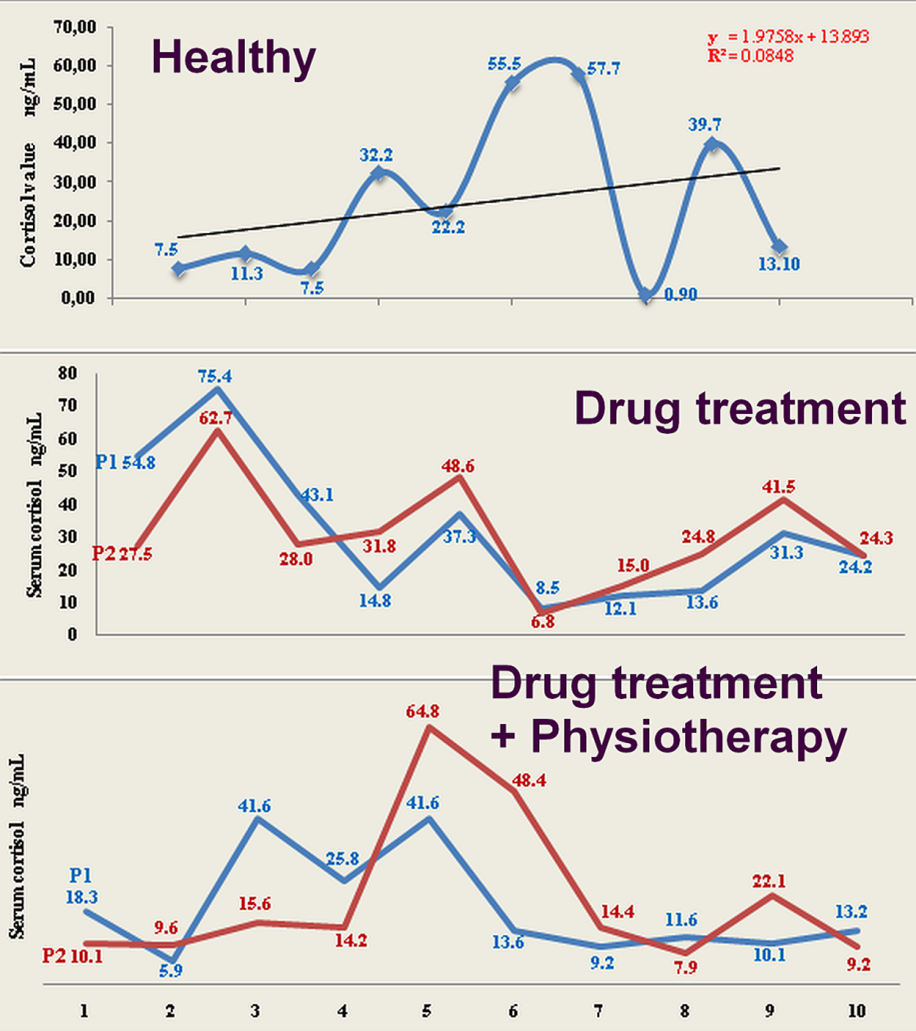

Supplement: Supplementary file 1 [file animals-10-02036-s001.zip › animals-975805-supplementary.png]
